# Osteochondrogenesis with Autologous Peripheral Blood Stem Cells for Osteochondral Lesions of the Talus: Report of Five Cases

**DOI:** 10.5704/MOJ.2211.019

**Published:** 2022-11

**Authors:** KY Saw, CSY Jee, A Ramlan, A Dawam, YC Saw, SF Low

**Affiliations:** 1 Department of Orthopaedic Surgery, Kuala Lumpur Sports Medicine Centre, Kuala Lumpur, Malaysia; 2 Stem Cells Research and Development, Kuala Lumpur Sports Medicine Centre, Kuala Lumpur, Malaysia; 3 Department of Radiology, Kuala Lumpur Sports Medicine Centre, Kuala Lumpur, Malaysia

**Keywords:** osteochondral lesions of the talus, ankle osteoarthritis, osteochondrogenesis, peripheral blood stem cells, marrow stimulation

## Abstract

Osteochondral lesions of the talus (OLTs) may progress to ankle arthritis needing ankle arthroplasty or arthrodesis. We report five cases of OLTs treated along the principles developed for chondrogenesis of the knee joint with autologous peripheral blood stem cells (PBSCs), resulting in repair and regeneration of the bone and cartilage components. Improvement in Ankle Osteoarthritis Scale (AOS) scores with minimum two years follow-up showed statistical significance (p < 0.05).

## Introduction

Osteochondral lesions of the talus (OLTs) involve injury to the ankle articular cartilage and the underlying subchondral bone, often associated with ankle pain and dysfunction. Left untreated, OLTs risk progression to ankle arthritis needing ankle arthroplasty or arthrodesis.

We developed arthroscopic K.A.R.T. (KLSMC Articular Regeneration Technology) to address massive knee chondral defects^1^ and end-staged ankle arthritis^2^. Applying the same principles developed for chondrogenesis with autologous peripheral blood stem cells (PBSCs), we report the results of applying K.A.R.T. to address OLTs by repair and regeneration of the bone and cartilage components.

## Case Reports

**Fig. 1.**
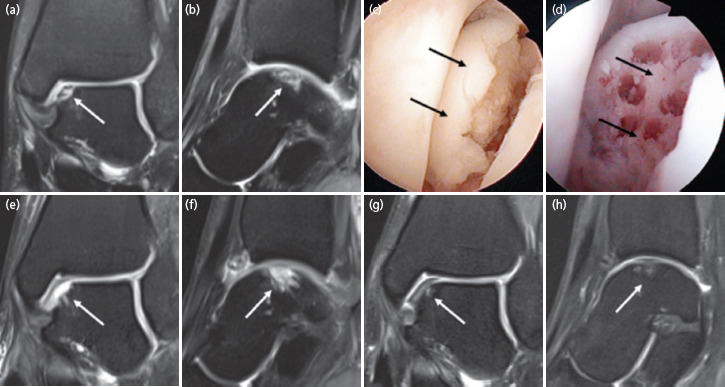
(a) Coronal and (b) sagittal MRI of the left ankle showing osteochondral lesion at the medial aspect of the talar dome (white arrows) from case 1. (c) Arthroscopic view over the medial aspect of the left ankle joint, showing the loose osteochondral fragment (black arrows). (d) View after removal of the osteochondral fragment followed by subchondral drilling (black arrows). MRI of the left ankle performed one day after surgery, with (e) coronal and (f) sagittal views following removal of the loose osteochondral fragment and subchondral drilling (white arrows) without bone grafting. Corresponding images at 2 years, (g) and (h) showing repair and regeneration of the bone and cartilage components (white arrows). These are fat-suppressed proton density-weighted MR images.

The first case was a 17-year-old female teenager presented with a 3 years’ history of left ankle pain following an injury sustained at ballet. The pain was aggravated by prolonged walks and running. MRI scan (Fig. 1a, 1b) revealed an osteochondral lesion of the medial talar dome measuring 0.9cm length (L) x 0.5cm width (W) x 0.8cm depth (D). Arthroscopic removal of the loose osteochondral fragment was performed followed by subchondral drilling into the defect (Fig. 1c, 1d) as previously described^1^-^3^.

One week after surgery, PBSCs were harvested. The details of the harvesting procedure and cell preparation are outlined in our previous publication^3^. Immediately after the apheresis process, 8ml fresh PBSCs aliquot was mixed with 2ml hyaluronic acid (HA) and injected into the operated ankle joint under aseptic conditions. Haemarthrosis was aspirated prior to each injection. At 4 subsequent weekly intervals, an 8ml aliquot of the thawed cryopreserved PBSCs mixed with 2ml HA were injected into the operated ankle joint. Physiotherapy with joint mobilisation commenced one day after surgery. Partial weight bearing progressed from day one after surgery to full weight bearing in six weeks. At month 6, 12, 18 and 24 following surgery, 3 additional weekly intra-articular injections comprising 4ml thawed cryopreserved PBSCs and 2ml HA were given. MRI of the left ankle performed one day after surgery (Fig. 1e, 1f) showed the removed loose osteochondral fragment and subchondral drilling without bone grafting. Corresponding images at two years, (Fig. 1g, 1h) showed osteochondral regeneration and integration. Patient returned to sporting activities at two years after surgery.

**Fig. 2.**
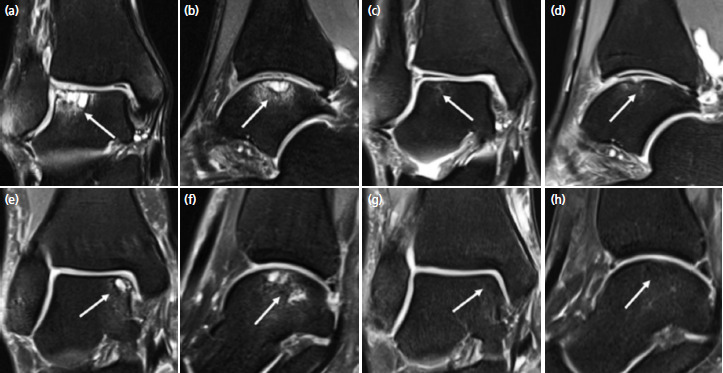
(a) Coronal and (b) sagittal MR images of the second case showing right ankle osteochondral lesion at the lateral aspect of the talar dome (white arrows). Corresponding images at 2 years, (c) and (d) showing osteochondral regeneration and integration (white arrows). MR images of the third case with coronal (e) and (f) sagittal views of the right ankle showing osteochondral lesion at the medial aspect of the talar dome (white arrows). Corresponding images at 2 years, (g) and (h) showing satisfactory healing. These are all fat-suppressed proton density-weighted MR images.

Second case was a 33-year-old man who presented with a 4 years’ history of right ankle pain following recurrent ankle sprains from basketball. MRI scan (Fig. 2a, 2b) revealed an osteochondral lesion of the lateral talar dome measuring 1.2cm (L) x 1.3cm (W) x 0.8cm (D). Osteochondral regeneration is clearly observed from MRI images (Fig. 2c, 2d) at two years after surgery.

Third case was a 50-year-old woman who sustained a skiing injury and presented with a 4 years’ history of right ankle pain. MR images of her right ankle (Fig. 2e, 2f) revealed an osteochondral lesion of the medial talar dome measuring 0.7cm (L) x 0.5cm (W) x 0.5cm (D). MR images at two years (Fig. 2g, 2h) showed satisfactory healing.

The fourth (35-year-old man) and fifth (51-year-old man) cases presented with medial talar dome OLTs of the right ankle measuring 1.0cm (L) x 0.7cm (W) x 0.9cm (D) and 1.4cm (L) x 0.6cm (W) x 1.0cm (D), respectively.

The surgical procedures and post-operative management of the above cases were similar to the first case. Written informed consent was taken from all cases reported here. There were no documented infection or major adverse events.

## Discussion

The results of this case series showed the ability of K.A.R.T. to repair and regenerate both the articular cartilage and the underlying subchondral bone of OLTs with clinical and radiological improvements, supporting the application of PBSCs for osteochondrogenesis.

Treatment options for OLTs include marrow stimulation procedures with or without biologics, osteochondral autograft / allograft transfer system (OATS) and Matrix-induced autologous chondrocyte implantation (MACI).

Powers *et al* looked at various treatment options for OLTs^4^. Bone marrow stimulation following debridement and curettage may be considered in lesions with a diameter less than 10mm, with surface area less than 100mm^2^, and a depth less than 5mm. They also suggested augmentation with cancellous bone graft for subchondral cysts with volume greater than 100mm^3^ and mentioned that lesions greater than 1.29cm^2^ as in our Case 2 (1.56cm^2^) were potential candidates for OATS. In our case series, the depth of all the lesions was ≥ 5mm and therefore not suitable for bone marrow stimulation procedure alone. We calculated each lesion's volume by assuming a cylinder lesion shape and averaging the length and width for an average diameter. The average volume of our 5 cases was 545mm^3^ with Case 3 having the smallest lesion at 141mm^3^ and Case 2 with the largest lesion at 981mm^3^. Therefore, theoretically all the 5 cases reported here should potentially be augmented with cancellous bone graft.

MRI scans of our case series with images at two years (Fig. 1, 2) showed satisfactory repair and regeneration of the bone and cartilage components following K.A.R.T. procedure. There were no evidence of residual cystic lesions or delamination of the overlying articular cartilage. The scans revealed no evidence of adverse articular or extra-articular abnormalities.

**Fig. 3.**
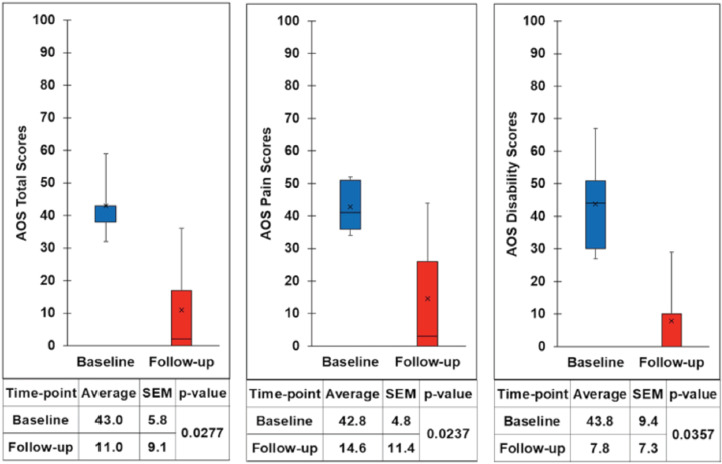
Box plot showing clinical outcome measure scores, pre-operatively and at the time of the latest follow-up for the five cases, with a minimum two years follow-up. The top and bottom of the box represent the interquartile range, the line within the box represents the median, the ‘x’ within the box represents the mean and the whiskers indicate the range. Ankle Osteoarthritis Scale (AOS) scores show greater pain and disability when the number is higher. Improvement of pain and disability (p < 0.05) is indicated by lower numbers at follow-up. SEM = Standard Error of Mean.

The Ankle Osteoarthritis Scale (AOS) scores of the reported five cases are presented (Fig. 3, Table I). This showed improvement in clinical outcome measure scores (mean difference between the baseline to final follow-up scores). Our mean improvement for the AOS pain (28) and AOS disability (36) with p < 0.05 are comparable to those reported by Vannini *et al* (AOS pain 34 and AOS disability 35)^5^.

**Table I:** Patient demographics and improvement in clinical outcome measure scores from baseline to final follow-up in all five cases

					Clinic outcome measure scores improvement		
Case	Gender	Age at surgery (years)	BMI at surgery (kg/m^2^)	Period of follow-up (years)	AOS total	AOS pain	AOS disability
1	Female	17	21.8	3.7	26	15	34
2	Male	33	24.2	7.0	30	31	30
3	Female	50	26.9	2.8	43	36	51
4	Male	35	26.9	10.0	59	52	67
5	Male	51	23.4	6.5	2	7	-2
Mean	-	37	24.7	6.0	32	28	36

We recently reported the ability of K.A.R.T. to address end-staged ankle arthritis^2^ as treatment failure of OLTs leads to persistent pain and progressive arthritis. This case report supports the use of K.A.R.T. for OLTs without the need for bone grafting or more complex procedures like MACI and OATS which may involve malleolar osteotomy. This showed the ability of this technology to reverse the pathology and hence arrest or slow down the degenerative changes associated with OLTs, potentially avoiding more complicated procedures for example joint arthroplasty or arthrodesis to address advanced ankle arthritis.

Arthroscopic subchondral drilling followed by postoperative intra-articular injections of autologous PBSCs plus HA is effective for the management of OLTs.
